# A case report of retroperitoneal liposarcoma

**DOI:** 10.1097/MD.0000000000039633

**Published:** 2024-09-13

**Authors:** Zicheng Bao, Zhidong Zhang, Pingan Ding, Qun Zhao, Yong Li

**Affiliations:** a Third Department of Surgery, Fourth Hospital of Hebei Medical University, Shijiazhuang, China.

**Keywords:** case report, leukocytosis, retroperitoneal liposarcoma

## Abstract

**Background:**

Retroperitoneal liposarcoma is a rare and complex tumor originating from the mesenchymal tissues, with no specific manifestations in the early stage, and a large tumor size in the late stage. Patients often consult a physician because of large abdominal mass, increased abdominal circumference, and abdominal pain, and rarely because of leukocytosis.

**Patient concerns:**

A 54-year-old female presented to our hospital with complaints of “abdominal distension for over 3 months, left lumbar pain for over 2 months.” Considering the comprehensive symptoms, examinations, computed tomography scans, and pathological results, the possibility of retroperitoneal liposarcoma is high.

**Diagnoses:**

Retroperitoneal liposarcoma with leukocytosis.

**Interventions:**

Open retroperitoneal mass excision along with transcystoscopic left ureteral Double-J Ureterl Stent Insertion tube placement and left nephrectomy.

**Outcomes:**

The postoperative pathological findings of the abdominal mass, combined with morphological and immunohistochemical results, are consistent with retroperitoneal liposarcoma. The patient had no recurrence in 7 months of postoperative follow-up conducted on the telephone and is now in continued follow-up.

**Conclusion:**

Retroperitoneal liposarcoma is highly malignant and prone to recurrence. Radical surgery is currently the primary treatment modality for patients with this condition. Analogous to cancer patients, those with elevated white blood cell counts and retroperitoneal liposarcoma may have poor prognoses, with a high likelihood of local recurrence and distant metastasis. Close postoperative follow-up is necessary. Therefore, regular postoperative review of blood routine may be a relatively economical and convenient method for the early detection of recurrence and metastasis of retroperitoneal liposarcoma.

## 1. Introduction

Retroperitoneal liposarcoma is clinically asymptomatic in the early stages of development, and early detection of these tumors involves the use of various imaging tests. The main screening modality is ultrasound, whereas the preferred modality is computed tomography (CT) and magnetic resonance imaging. CT scanning, enhancement, three-dimensional reconstruction, and CT angiography can accurately assess the proximity of the tumor to abdominal organs and vessels, which is important for planning the surgical approach while considering the associated risks. Liposarcomas have diverse magnetic resonance imaging and CT appearances due to the various subtypes.^[1]^ The gold standard for differentiating between liposarcomas and other mesenchymal tumors is the pathological examination. Pathology can be obtained by ultrasound-guided or CT-guided puncture. Currently, surgical resection remains the preferred treatment option for retroperitoneal fat tumors, as retroperitoneal liposarcomas are less responsive to chemotherapy and radiotherapy as compared to other tumors, and the effectiveness of targeted therapies and immunotherapies in the treatment of retroperitoneal liposarcomas remains a subject of research and debate.

## 2. Case introduction

**Figure 1.**
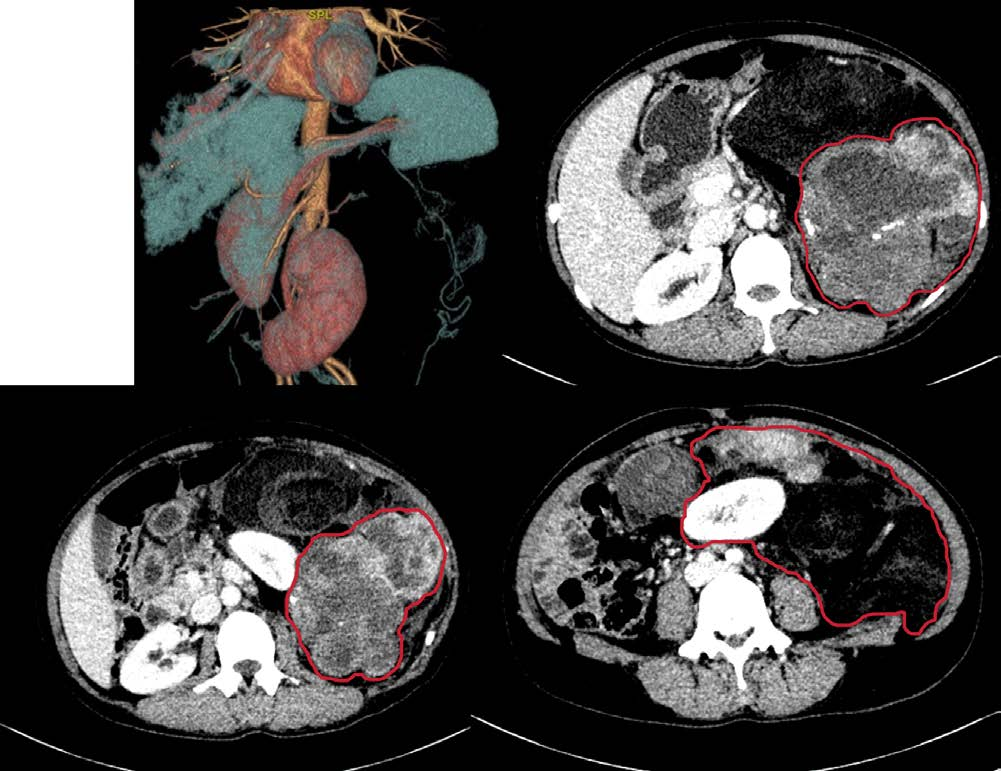
Contrast-enhanced CT. CT = computed tomography.

**Figure 2.**
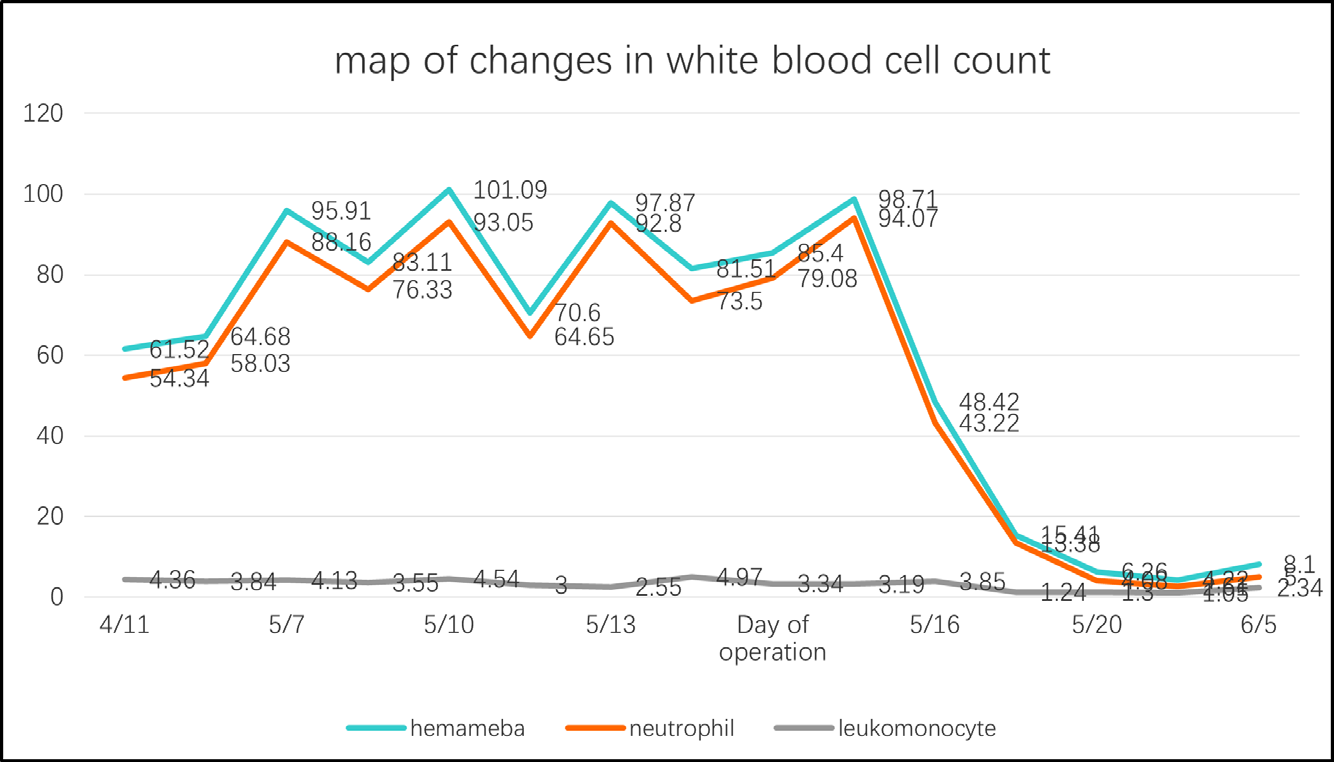
Map of changes in white blood cell count.

A 54-year-old woman with abdominal distension for >3 months and left lumbar distension for >2 months presented to the Fourth Hospital of Hebei Medical University in May 2023, and was found to have a large mass from the rib margin down to the pubic symphysis. The patient did not receive any therapy and had no significant family history. Abdominal examination revealed a large mass from the lower rib margin to the pubic symphysis, with poor mobility and resistance to being pushed, and an abdominal circumference of 91 cm (Fig. 1). Abdominal enhancement CT showed a shadow of a mass in the posterior part of the spleen, with a size of about 12.7 × 11.8 × 14.2 cm, and abdominal organs being displaced by the compression. The CT revealed a shadow of multiple masses in the abdominal cavity, with a fat density, a solid component that does not enhance substantially, and peritoneal thickening. Pathological findings of the ultrasound-guided puncture showed hyperplastic fibrous tissue and a few actively growing spindle cell tumor-like tissues. The hematological examination after the admission to the hospital revealed a leukocyte count of 61.52 × 109/L. The preoperative leukocyte count was up to 101.09 × 109/L (Fig. 2). Findings of the bone marrow aspiration showed that peripheral blood nucleated cytometer neutrophils were predominantly neutrophilic rod-shaped and lobulated, mature erythrocytes were of different sizes, and small cells were seen in piles. Bone marrow flow cytology revealed that the proportion of granulocytes in the maturation stage was significantly increased, and no obvious abnormality was observed in the immunophenotype. Mutational testing of myeloproliferative tumor-related genes reported the following mutations as negative: JAK2V617F, JKA2 (exon12) gene N542-E543del, E543-D544del deletion, JKA2 (exon12) gene K539L1/L2 mutation, MPL (exon10) W515K/A/L/R1/R2/S mutation, MPL (exon10) S505N mutation, CALR gene (exon9) L367fs*46, and CALR gene (exon9) K385fs*47. The BCR-ABL fusion gene results were negative. Considering the current bone marrow biopsy, flow cytometry, and related genetic testing, chronic myeloid leukemia is not considered for the time being, but chronic neutrophilic leukemia cannot be ruled out, nor can paraneoplastic syndrome caused by abdominal tumors and leukemoid reactions be excluded. After a trial of oral hydroxyurea, the patient’s white blood cell count and neutrophil count did not show a significant decrease. Following a multidisciplinary treatment discussion, it was decided to proceed with surgical treatment.

**Figure 3.**
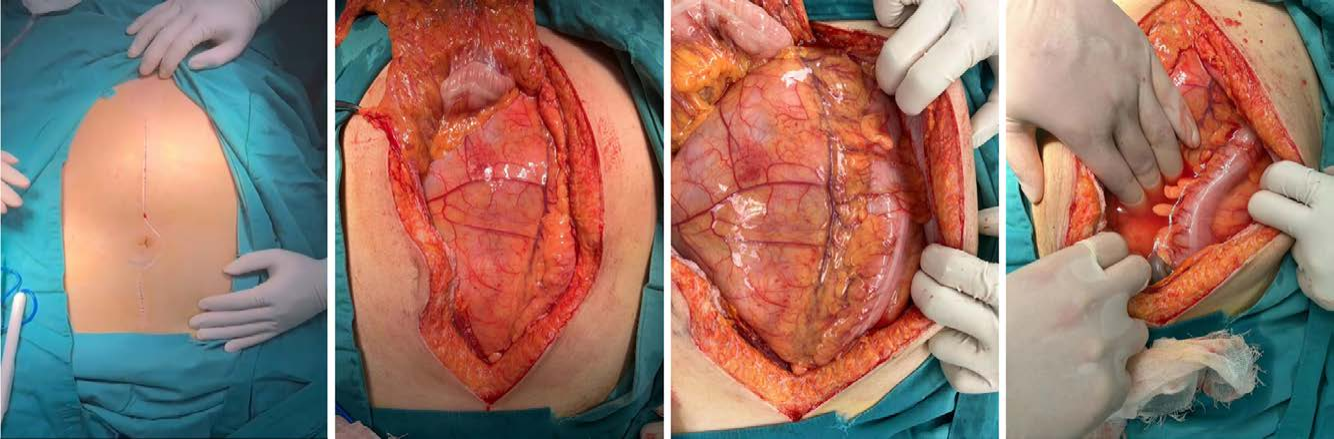
Intraoperative condition.

**Figure 4.**
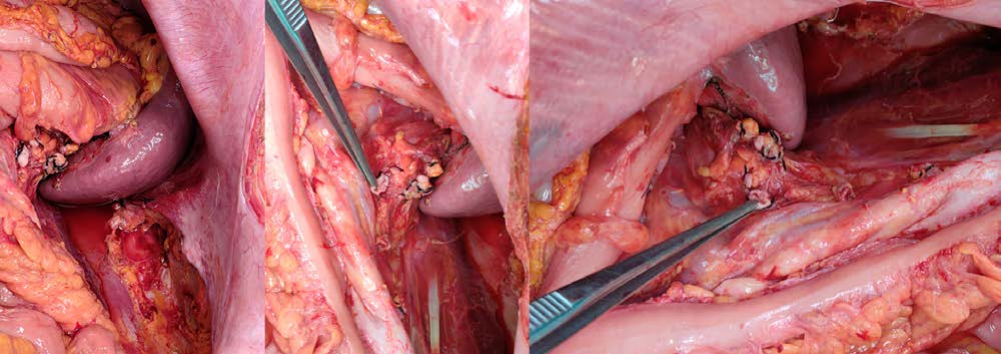
Intraoperative condition.

Assessing the patient’s clinical symptoms and findings of the relevant auxiliary examinations, the patient was considered to have a high possibility of a retroperitoneal liposarcoma, and therefore, open retroperitoneal mass excision along with transcystoscopic left ureteral Double-J Ureterl Stent Insertion tube placement and left nephrectomy were performed under general anesthesia on May 15, 2023. Intraoperative findings revealed that the mass was adherent to the omentum majus and transverse colonic mesentery, and the surface of the mass was rich in blood supply, with a size of approximately 40 × 25 × 15 cm, was brittle, had an intact envelope, and was irregular in shape (Figs. 3 and 4). After separating the mass from the peritoneum and transverse colon on both sides, it was observed that the small intestine and stomach were extruded to the right lower abdomen by the mass. Probing the abdominal pelvic floor and the adhesion of the mass, the patient was considered to have originated from the left renal hilar region, the mass completely wrapped the left kidney, and the mass was resected together with the left kidney.

**Figure 5.**
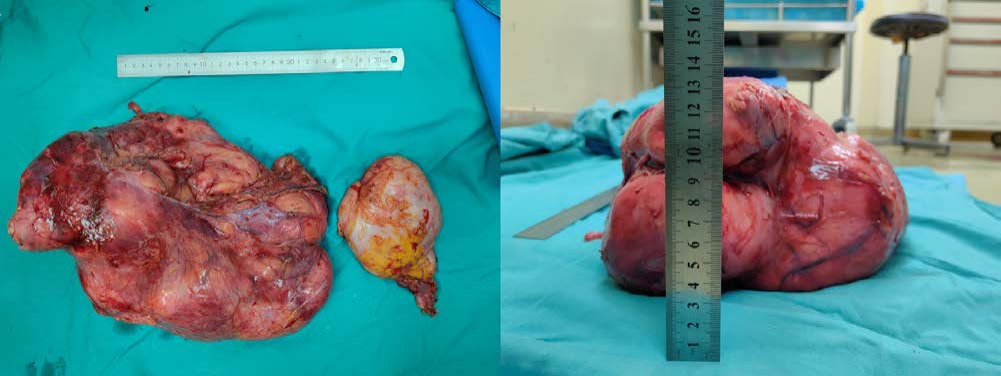
Postoperative specimen.

**Figure 6.**
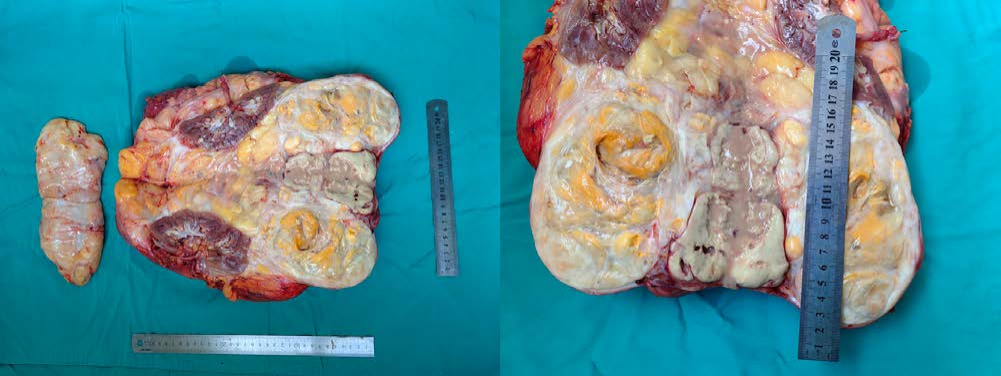
Gross photo of postoperative specimen.

**Figure 7.**
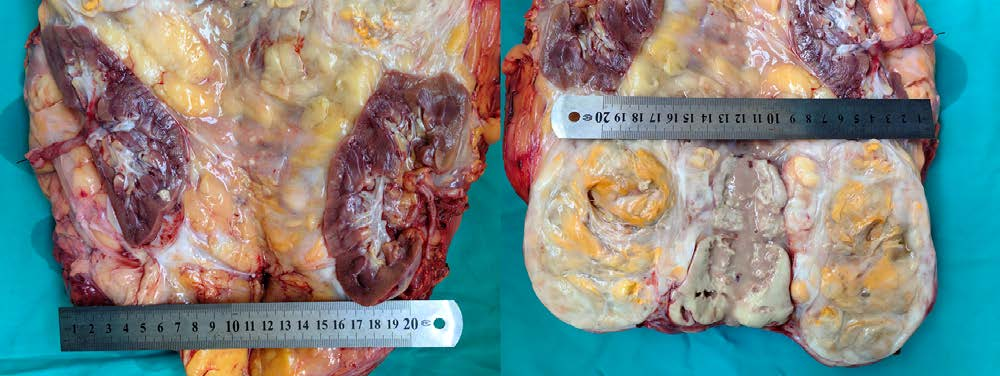
Gross photo of postoperative specimen.

**Figure 8.**
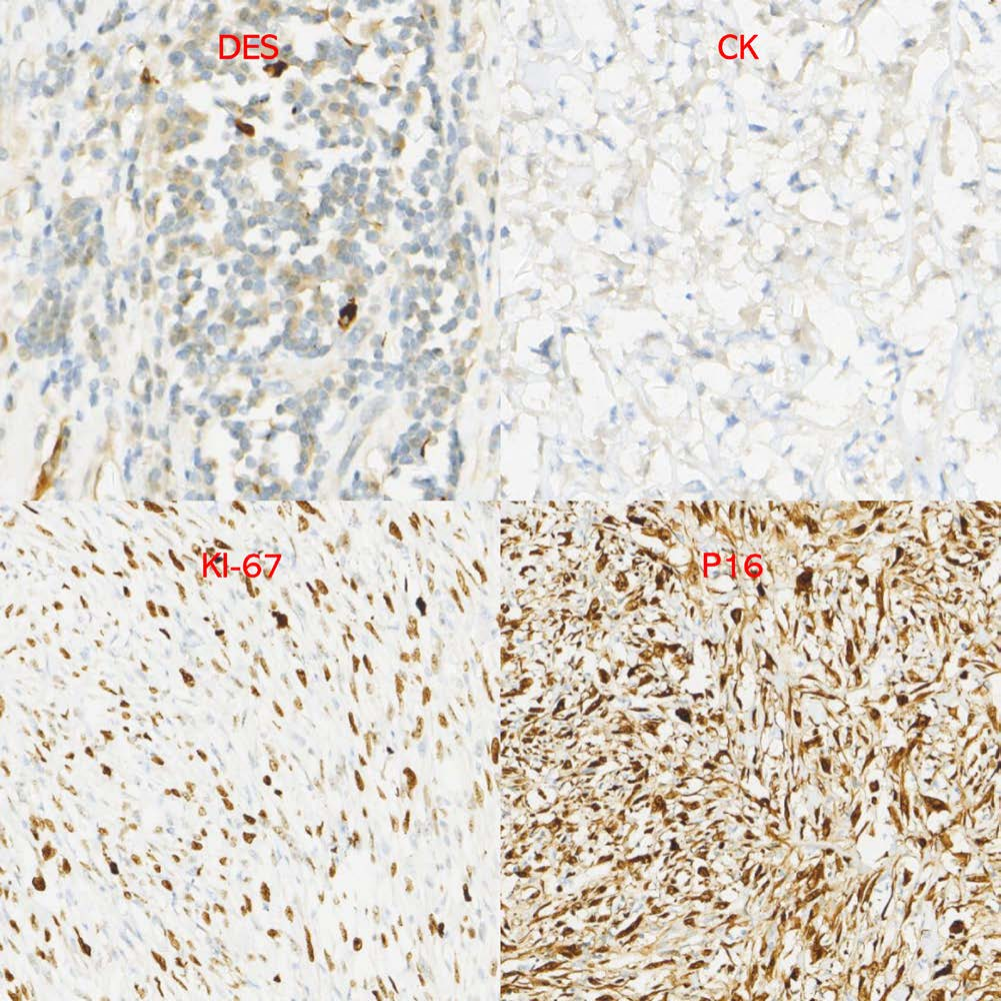
Postoperative pathology immunohistochemistry.

**Figure 9.**
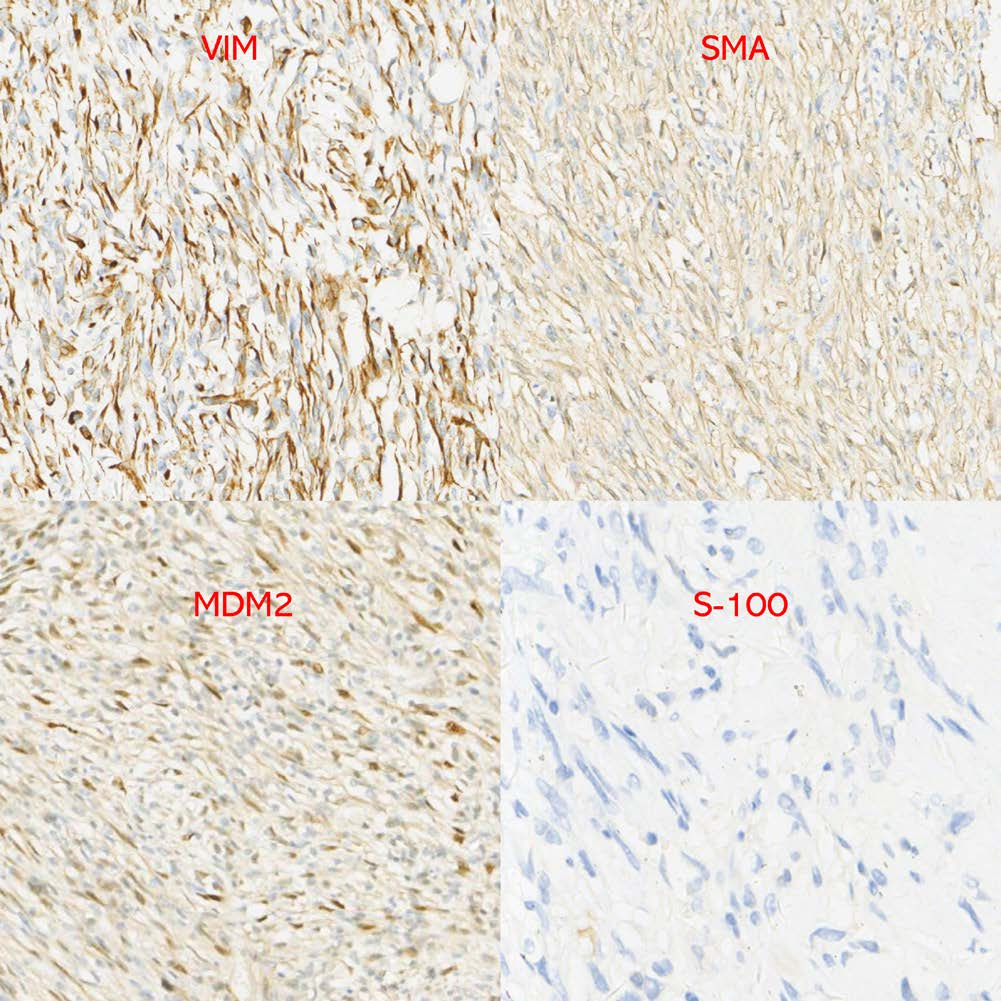
Postoperative pathology immunohistochemistry.

Postoperative routine pathology revealed 2 masses, one of them 35 × 32 × 7 cm, within which visible kidney tissue 13 × 7 × 6 cm, mass incision cut surface grayish yellow texture tough, part of the area jelly-like, and visible necrosis (Figs. 5–7). A tumor of mesenchymal origin was seen outside the renal tissue, indicating a liposarcoma, and immunohistochemical results are pending further diagnosis. The second mass was 20 × 10 × 4 cm, a section of gray–white–gray–yellow texture, tough, mesenchymal origin tumor, indicating a liposarcoma and immunohistochemical results are pending further diagnosis. Immunohistochemistry results revealed the following: AE1/AE3 (−), vimentin (+), S100 (−), MDM2 (+), P16 (+), Des (−/+), SMA (−/+), and Ki67 (30% positive cells) (Figs. 8 and 9). Considering dedifferentiated liposarcoma, further fluorescence in situ hybridization testing was recommended. CDK4 gene fluorescence in situ hybridization results were positive and showed CDK4 gene amplification. The patient had no recurrence in 7 months of postoperative follow-up conducted on the telephone and is now in continued follow-up.

## 3. Discussion

Retroperitoneal liposarcoma is a soft tissue malignant tumor of adipose tissue origin that occurs in the retroperitoneal space and accounts for approximately 45% of all primary retroperitoneal soft tissue sarcomas.^[2–4]^ The global annual prevalence is approximately 3 to 4 cases per million,^[5]^ and the disease can occur in all age groups, with a peak incidence in the 40s and 60s and a slightly higher prevalence in males than in females. Patients with retroperitoneal liposarcoma are often diagnosed with abdominal distension, abdominal pain, and other symptoms caused by compression of abdominal organs by a large abdominal mass, which is difficult to diagnose and treat because of the large size of the tumor, complex anatomical relationship, and abundant blood supply. In a retrospective study, Taguchi et al^[6]^ showed that clinical symptoms at the time of diagnosis were an independent risk factor for progression-free survival.

The modality of choice for diagnosing liposarcomas is CT. CT plain + enhancement + three-dimensional reconstruction, CT angiography can reveal the proximity of the tumor to the abdominal organs and large abdominal vessels. Bhosale et al^[5]^ analyzed the findings of abdominal CT in 49 patients with untreated liposarcoma in a retrospective study and concluded that there was a correlation between the imaging characteristics of the patients and the postoperative pathology findings. In highly differentiated liposarcoma, nodules with enhancement or central necrosis are highly likely to be dedifferentiated components.

Currently, liposarcomas are not responsive to chemotherapy and radiotherapy and the effectiveness of targeted therapies and immunotherapies is debatable, therefore surgical resection is the preferred treatment option regardless of whether it is a first occurrence or a recurrent tumor and complete removal of the tumor is the primary goal of the treatment strategies. Sogaard et al^[7]^ retrospectively analyzed the 5-year survival rate of 73 patients with liposarcoma. The 5-year survival rate was 70.2% for primary liposarcoma and 51.8% for first recurrent sarcoma. However, the 5-year survival rate for patients undergoing radical surgery exceeded 70% in both the primary and recurrent groups. Retroperitoneal liposarcoma has a high recurrence rate, up to 85% for the dedifferentiated type, and the interval between recurrences is progressively shorter, with a major proportion of patients experiencing multiple recurrences and requiring multiple surgeries. Approximately 75% of the patients die from multiple recurrences. High recurrence is the current difficulty in the treatment of retroperitoneal liposarcomas.

Lee et al^[8]^ concluded that most retroperitoneal liposarcomas originate from the kidneys, and therefore renal involvement is often present in patients. Owing to the growth or compression of the tumor, it often results in the displacement of the kidney or complete encapsulation of the kidney. Complete surgical removal of the tumor is the most important part of treatment in these patients. Even if combined organ resection is required, it is important to remove the tumor as completely as possible as this improves the chances of patient survival and reduces the likelihood of recurrence. The incidence of histopathological organ invasion in retroperitoneal sarcomas has also been suggested by Fairweather et al^[9]^ to influence the patient’s 5-year overall survival and recurrence rate. In this case, the patient had no previous history of related tumors, intraoperative exploration showed that the mass was brittle, with intact peritoneum, irregular in shape, and adherent to the greater omentum and transverse colonic mesentery; most of the area was severely adherent, with a rich blood supply on the surface of the mass; the size of the mass was approximately 40 × 25 × 15 cm, the abdominal pelvic floor was adherent to the mass, and the mass was found to have originated from the left renal hilar region along the root exploration and completely encapsulated the left kidney. Therefore, to achieve the complete effects of radical resection, the left kidney was removed completely.

The striking difference between the patient and other patients with retroperitoneal tumors is the presence of abnormally high leukocytes. Based on the bone puncture results, leukemia can be excluded. Some researchers have proposed a “leukemia-like reaction” for patients with malignant tumors accompanied by increased leukocytes, that is, patients with advanced malignant tumors have peripheral blood leukocytes of >50 × 109/L, 90%–46% of neutrophils, naïve granulocytes in the peripheral blood with obvious nuclear shift, and toxic granulocytes with vacuoles and toxic granulocytes degeneration. Leukemia-like reactions can be caused by infection, allergy, malignant tumor, etc. Leukemia-like reactions are often indicative of advanced tumor stage, critical condition, and poor prognosis. Some studies have found that the degree of leukocyte elevation correlates with poor prognosis. Analogous to the leukocytosis present in patients with cancer, there may be 2 reasons for the elevated leukocytes in patients with retroperitoneal tumors. First, the tumor itself produces and releases granulocyte colony-stimulating factor, which stimulates bone marrow hematopoiesis, particularly causing an increase in granulocytes. Second, necrosis of the tumor tissue, and the resulting breakdown products, may stimulate the release of bone marrow neutrophils, thus contributing to leukocytosis. Scott et al^[10]^ shared a case of a patient with advanced hyperdifferentiated liposarcoma that converted to dedifferentiated liposarcoma after 3 cycles of eribulin with severe leukocytosis. Although the likelihood of bone marrow metastasis is low in such patients, abnormal leukocytosis accompanied by an increased granulocyte colony-stimulating factor should prompt a bone marrow biopsy to rule out bone marrow metastasis in the patient. It has been shown that surgery and storage of the primary tumor can reactivate dormant disseminated tumor cells, leading to metastasis and rapid disease progression.

## 4. Conclusion

Retroperitoneal liposarcoma is considered highly malignant and has a high recurrence rate. CT and ultrasound-guided puncture aid in the preoperative diagnosis and evaluation of patients. Currently, radical surgery is the main treatment of choice for these patients, as the effectiveness of radiotherapy for these patients is a matter of debate, and no targeted drug is available. Similar to patients with cancers, patients with accompanying leukocytosis in retroperitoneal liposarcoma may have a poor prognosis, with a high likelihood of local recurrence and distant metastasis, and require a close postoperative follow-up. Although surgical resection can temporarily solve the short-term problems of patients with retroperitoneal liposarcoma, it is necessary to explore ways to supplement radical surgical resection with effective chemotherapy, immunotherapy, targeted therapy or radiation therapy, or even perioperative peritoneal perfusion therapy to reduce the likelihood of recurrence or metastasis and to consolidate the efficacy of the surgical treatment. Leukemoid reaction often occurs in cancer patients. Currently, there is still relatively little related literature on retroperitoneal liposarcoma and leukemoid reaction, and there is also a lack of analysis and collation of a large amount of clinical data. Therefore, the relationship between the 2 still needs further demonstration. Whether regular reexamination of blood routine can directly confirm the recurrence and metastasis of patients awaits the support of a large amount of clinical data.

## Author contributions

**Conceptualization:** Zicheng Bao, Zhidong Zhang.

**Formal analysis:** Zicheng Bao, Pingan Ding.

**Investigation:** Zicheng Bao, Zhidong Zhang, Pingan Ding, Yong Li.

**Methodology:** Zicheng Bao, Pingan Ding, Yong Li.

**Resources:** Zicheng Bao, Zhidong Zhang.

**Writing—original draft:** Zicheng Bao.

**Writing—review & editing:** Zicheng Bao, Zhidong Zhang, Pingan Ding, Qun Zhao.

**Funding acquisition:** Zhidong Zhang, Yong Li.

**Project administration:** Zhidong Zhang, Qun Zhao.

**Supervision:** Zhidong Zhang, Qun Zhao.

**Validation:** Zhidong Zhang.
